# Supplementary material for: Early prediction of in-hospital death of COVID-19 patients: a machine-learning model based on age, blood analyses, and chest x-ray score
Source: eLife. 2021 Oct 18;10:e70640. doi: 10.7554/eLife.70640 (PMC8550757; doi:10.7554/eLife.70640)
Supplement: Supplementary file 1. — (a) Descriptive statistics on all variables of the entire sample. (b) Descriptive statistics on all variables in the dataset stratified respect first (March–April 2020) and second (May–December 2020) wave. Comparison between alive and dead. (c): Performance metrics of the random forest (RF) using or not a rebalanced dataset with the synthetic minority oversampling technique (SMOTE) methodology. In this table we compare the performance of two RFs applied on (i) a dataset rebalanced with the SMOTE methodology and (ii) the original dataset. This analysis suggests the use of SMOTE methodology before applying RF since the performance in training and validating groups (especially in terms of sensitivity) are better respect those obtained from the RF grown on the original dataset. (d): Performance metrics of the random forests (RFs) estimated on single biomarkers. (e): Optimal threshold for each biomarker to predict the outcome. [file elife-70640-supp1.docx]

**Supplementary File 1**

**Supplementary File 1a**

| **Variables** | **Overall (N=2782)** |
| --- | --- |
| **Age** |  |
| Mean (SD) | 66.84 (14.44) |
| Median (Q1, Q3) | 69.00 (57.00, 78.00) |
| Range | 18.00 - 98.00 |
| **Sex** |  |
| F | 1010 (36.3%) |
| M | 1772 (63.7%) |
| **Days in hospital** |  |
| N-Miss | 96 |
| Mean (SD) | 13.96 (11.60) |
| Median (Q1, Q3) | 11.00 (6.00, 18.00) |
| Range | 0.00 - 140.00 |
| **Score** |  |
| Mean (SD) | 6.97 (4.52) |
| Median (Q1, Q3) | 7.00 (3.00, 10.00) |
| Range | 0.00 - 18.00 |
| **D-dimer** |  |
| N-Miss | 663 |
| Mean (SD) | 1638.18 (4218.89) |
| Median (Q1, Q3) | 578.00 (315.50, 1289.50) |
| Range | 190.00 - 60342.00 |
| **Fibrinogen** |  |
| N-Miss | 518 |
| Mean (SD) | 528.61 (191.92) |
| Median (Q1, Q3) | 515.00 (391.00, 637.00) |
| Range | 68.00 - 1371.00 |
| **LDH** |  |
| N-Miss | 348 |
| Mean (SD) | 337.48 (251.21) |
| Median (Q1, Q3) | 291.50 (226.00, 395.00) |
| Range | 90.00 - 6689.00 |
| **Neutrophils** |  |
| N-Miss | 47 |
| Mean (SD) | 5.96 (3.87) |
| Median (Q1, Q3) | 5.03 (3.43, 7.48) |
| Range | 0.00 - 53.99 |
| **Lymphocytes** |  |
| N-Miss | 47 |
| Mean (SD) | 1.35 (4.66) |
| Median (Q1, Q3) | 1.00 (0.69, 1.40) |
| Range | 0.04 - 177.63 |
| **Neutrophils on Lymphocytes** |  |
| N-Miss | 47 |
| Mean (SD) | 7.27 (8.41) |
| Median (Q1, Q3) | 4.89 (2.96, 8.69) |
| Range | 0.00 - 143.25 |
| **Neutrophils %** |  |
| N-Miss | 46 |
| Mean (SD) | 0.74 (0.13) |
| Median (Q1, Q3) | 0.76 (0.67, 0.84) |
| Range | 0.00 - 0.99 |
| **Lymphocytes %** |  |
| N-Miss | 46 |
| Mean (SD) | 0.17 (0.11) |
| Median (Q1, Q3) | 0.16 (0.10, 0.23) |
| Range | 0.01 - 0.99 |
| **PCR** |  |
| N-Miss | 80 |
| Mean (SD) | 81.30 (81.30) |
| Median (Q1, Q3) | 56.70 (18.20, 117.55) |
| Range | 0.30 - 593.80 |
| **WBC** |  |
| N-Miss | 45 |
| Mean (SD) | 7.97 (6.65) |
| Median (Q1, Q3) | 6.82 (5.00, 9.40) |
| Range | 0.32 - 191.02 |
| **Basophils** |  |
| N-Miss | 47 |
| Mean (SD) | 0.02 (0.03) |
| Median (Q1, Q3) | 0.01 (0.01, 0.03) |
| Range | 0.00 - 0.84 |
| **Basophils %** |  |
| N-Miss | 46 |
| Mean (SD) | 0.00 (0.00) |
| Median (Q1, Q3) | 0.00 (0.00, 0.00) |
| Range | 0.00 - 0.06 |
| **Eosinophils** |  |
| N-Miss | 47 |
| Mean (SD) | 0.05 (0.12) |
| Median (Q1, Q3) | 0.01 (0.00, 0.06) |
| Range | 0.00 - 2.19 |
| **Eosinophils %** |  |
| N-Miss | 46 |
| Mean (SD) | 0.01 (0.02) |
| Median (Q1, Q3) | 0.00 (0.00, 0.01) |
| Range | 0.00 - 0.27 |
| **Monocytes** |  |
| N-Miss | 47 |
| Mean (SD) | 0.58 (1.39) |
| Median (Q1, Q3) | 0.47 (0.31, 0.68) |
| Range | 0.01 - 66.34 |
| **Monocytes %** |  |
| N-Miss | 46 |
| Mean (SD) | 0.08 (0.04) |
| Median (Q1, Q3) | 0.07 (0.05, 0.10) |
| Range | 0.00 - 0.72 |

**Supplementary File 1b**

|  | **Alive** | |  | **Dead** | |  |
| --- | --- | --- | --- | --- | --- | --- |
| **Variables** | **March-April (MA)**  **(N=1683)** | **May-December (MD)**  **(N=594)** | **p-value** | **March-April (MA)**  **(N=423)** | **May-December (MD)**  **(N=82)** | **p value** |
| **Age** |  |  | 0.1503* |  |  | 0.2041* |
| Mean (SD) | 64.55 (14.27) | 65.30 (15.20) |  | 76.21 (9.12) | 76.72 (10.79) |  |
| Median     (Q1, Q3) | 65.00  (55.00, 75.00) | 67.00  (55.00, 77.00) |  | 77.00  (72.00, 82.00) | 80.00  (72.25, 84.75) |  |
| Range | 19.00 - 97.00 | 18.00 - 97.00 |  | 44.00 - 98.00 | 44.00 - 98.00 |  |
| **Sex** |  |  | 0.0936** |  |  | 0.8968** |
| F | 613 (36.4%) | 240 (40.4%) |  | 131 (31.0%) | 26 (31.7%) |  |
| M | 1070 (63.6%) | 354 (59.6%) |  | 292 (69.0%) | 56 (68.3%) |  |
| **Days in hospital** |  |  | 0.2122* |  |  | ***< 0.001**** |
| N-Miss | 1 | 95 |  | 0 | 0 |  |
| Mean (SD) | 14.15 (11.66) | 14.95 (11.67) |  | 11.33 (10.98) | 17.77 (10.75) |  |
| Median     (Q1, Q3) | 11.00  (7.00, 18.00) | 12.00  (7.00, 20.00) |  | 8.00  (4.00, 15.00) | 17.50  (9.00, 25.00) |  |
| Range | 0.00 - 140.00 | 0.00 - 79.00 |  | 0.00 - 88.00 | 2.00 - 46.00 |  |
| **Score** |  |  | ***< 0.001**** |  |  | 0.3965* |
| Mean (SD) | 6.92 (4.40) | 5.65 (4.48) |  | 8.77 (4.39) | 8.23 (4.63) |  |
| Median     (Q1, Q3) | 7.00  (3.00, 10.00) | 5.00  (2.00, 9.00) |  | 9.00  (6.00, 12.00) | 9.00  (5.25, 11.00) |  |
| Range | 0.00 - 18.00 | 0.00 - 18.00 |  | 0.00 - 18.00 | 0.00 - 17.00 |  |
| **D-dimer** |  |  | ***< 0.001**** |  |  | ***0.0452**** |
| N-Miss | 406 | 128 |  | 113 | 16 |  |
| Mean (SD) | 1155.03 (2218.51) | 1538.17 (3123.38) |  | 3124.25 (8070.21) | 4712.44 (8897.82) |  |
| Median     (Q1, Q3) | 443.00  (262.00, 985.00) | 739.50  (427.50, 1341.25) |  | 944.50  (476.50, 2970.75) | 1112.00  (725.50, 3619.25) |  |
| Range | 200.00 - 47228.00 | 190.00 - 33501.00 |  | 200.00 - 60342.00 | 190.00 - 35000.00 |  |
| **Fibrinogen** |  |  | 0.7502* |  |  | 0.6711* |
| N-Miss | 339 | 54 |  | 117 | 8 |  |
| Mean (SD) | 530.53 (194.13) | 523.94 (169.43) |  | 530.55 (213.69) | 519.77 (213.05) |  |
| Median     (Q1, Q3) | 520.00  (381.00, 650.00) | 512.00  (405.00, 612.00) |  | 515.00  (381.00, 654.00) | 510.00  (330.50, 649.00) |  |
| Range | 119.00 - 1339.00 | 147.00 - 1371.00 |  | 68.00 - 1333.00 | 153.00 - 1287.00 |  |
| **LDH** |  |  | ***0.0298**** |  |  | ***0.0094**** |
| N-Miss | 188 | 61 |  | 92 | 7 |  |
| Mean (SD) | 321.25 (227.50) | 308.30 (196.23) |  | 433.71 (205.10) | 443.49 (707.95) |  |
| Median     (Q1, Q3) | 283.00  (222.00, 373.00) | 273.00  (218.00, 354.00) |  | 406.00  (269.50, 545.50) | 332.00  (257.00, 442.50) |  |
| Range | 90.00 - 6689.00 | 108.00 - 2565.00 |  | 123.00 - 1365.00 | 122.00 - 6310.00 |  |
| **Neutrophils** |  |  | 0.8529* |  |  | 0.6350* |
| N-Miss | 23 | 4 |  | 19 | 1 |  |
| Mean (SD) | 5.67 (3.61) | 5.80 (3.97) |  | 7.17 (4.39) | 7.21 (4.13) |  |
| Median     (Q1, Q3) | 4.83  (3.29, 7.03) | 4.78  (3.42, 7.11) |  | 6.20  (4.12, 9.02) | 6.72  (4.00, 9.77) |  |
| Range | 0.00 - 53.99 | 0.10 - 47.03 |  | 0.17 - 30.45 | 0.19 - 23.02 |  |
| **Lymphocytes** |  |  | 0.6287* |  |  | 0.2115* |
| N-Miss | 23 | 4 |  | 19 | 1 |  |
| Mean (SD) | 1.43 (5.48) | 1.22 (0.81) |  | 1.19 (4.29) | 1.38 (4.63) |  |
| Median  (Q1, Q3) | 1.04  (0.75, 1.42) | 1.06  (0.72, 1.52) |  | 0.81  (0.55, 1.18) | 0.74  (0.47, 1.06) |  |
| Range | 0.10 - 177.63 | 0.08 - 10.28 |  | 0.04 - 85.51 | 0.08 - 42.20 |  |
| **Neutrophils on Lymphocytes** |  |  | 0.8639* |  |  | 0.3122* |
| N-Miss | 23 | 4 |  | 19 | 1 |  |
| Mean (SD) | 6.18 (5.87) | 7.19 (9.92) |  | 10.72 (11.71) | 12.84 (13.09) |  |
| Median     (Q1, Q3) | 4.52  (2.84, 7.50) | 4.32  (2.63, 8.40) |  | 7.13  (4.47, 13.06) | 8.50  (4.05, 15.19) |  |
| Range | 0.00 - 101.90 | 0.12 - 143.25 |  | 0.01 - 129.67 | 0.11 - 70.56 |  |
| **Neutrophils %** |  |  | 0.6134* |  |  | 0.6882* |
| N-Miss | 22 | 4 |  | 19 | 1 |  |
| Mean (SD) | 0.73 (0.13) | 0.73 (0.13) |  | 0.80 (0.12) | 0.79 (0.16) |  |
| Median     (Q1, Q3) | 0.74  (0.66, 0.82) | 0.73  (0.64, 0.83) |  | 0.82  (0.75, 0.88) | 0.83  (0.69, 0.89) |  |
| Range | 0.00 - 0.97 | 0.10 - 0.99 |  | 0.01 - 0.97 | 0.10 - 0.96 |  |
| **Lymphocytes %** |  |  | 0.933* |  |  | 0.2924* |
| N-Miss | 22 | 4 |  | 19 | 1 |  |
| Mean (SD) | 0.18 (0.11) | 0.18 (0.11) |  | 0.13 (0.09) | 0.13 (0.13) |  |
| Median     (Q1, Q3) | 0.16  (0.11, 0.23) | 0.17  (0.10, 0.25) |  | 0.11  (0.07, 0.17) | 0.10  (0.06, 0.18) |  |
| Range | 0.01 - 0.97 | 0.01 - 0.88 |  | 0.01 - 0.99 | 0.01 - 0.88 |  |
| **PCR** |  |  | ***< 0.001**** |  |  | ***0.0235**** |
| N-Miss | 47 | 21 |  | 12 | 0 |  |
| Mean (SD) | 77.25 (75.76) | 64.28 (73.38) |  | 117.68 (95.97) | 98.59 (102.49) |  |
| Median      (Q1, Q3) | 55.65  (17.30, 111.60) | 39.10  (12.30, 91.10) |  | 99.20  (42.80, 170.45) | 74.80  (20.12, 140.73) |  |
| Range | 0.30 - 479.00 | 0.30 - 483.20 |  | 0.70 - 471.10 | 0.30 - 593.80 |  |
| **WBC** |  |  | 0.4686* |  |  | 0.6138* |
| N-Miss | 21 | 4 |  | 19 | 1 |  |
| Mean (SD) | 7.73 (7.13) | 7.65 (4.17) |  | 9.13 (7.46) | 9.23 (6.25) |  |
| Median     (Q1, Q3) | 6.62  (4.87, 9.11) | 6.67  (5.02, 8.90) |  | 7.62  (5.60, 10.74) | 8.34  (5.55, 12.04) |  |
| Range | 0.72 - 191.02 | 0.97 - 48.19 |  | 0.32 - 92.23 | 0.97 - 47.79 |  |
| **Basophils** |  |  | ***0.0013**** |  |  | 0.2253* |
| N-Miss | 23 | 4 |  | 19 | 1 |  |
| Mean (SD) | 0.02 (0.02) | 0.02 (0.04) |  | 0.02 (0.02) | 0.02 (0.02) |  |
| Median     (Q1, Q3) | 0.01  (0.01, 0.02) | 0.02  (0.01, 0.03) |  | 0.01  (0.01, 0.02) | 0.01  (0.01, 0.03) |  |
| Range | 0.00 - 0.31 | 0.00 - 0.84 |  | 0.00 - 0.15 | 0.00 - 0.11 |  |
| **Basophils %** |  |  | ***0.0012**** |  |  | 0.1017* |
| N-Miss | 22 | 4 |  | 19 | 1 |  |
| Mean (SD) | 0.00 (0.00) | 0.00 (0.00) |  | 0.00 (0.00) | 0.00 (0.00) |  |
| Median     (Q1, Q3) | 0.00  (0.00, 0.00) | 0.00  (0.00, 0.00) |  | 0.00  (0.00, 0.00) | 0.00  (0.00, 0.00) |  |
| Range | 0.00 - 0.02 | 0.00 - 0.05 |  | 0.00 - 0.06 | 0.00 - 0.01 |  |
| **Eosinophils** |  |  | 0.4863* |  |  | 0.9056* |
| N-Miss | 23 | 4 |  | 19 | 1 |  |
| Mean (SD) | 0.06 (0.12) | 0.06 (0.14) |  | 0.04 (0.10) | 0.05 (0.13) |  |
| Median     (Q1, Q3) | 0.01  (0.00, 0.07) | 0.01  (0.00, 0.06) |  | 0.00  (0.00, 0.02) | 0.00  (0.00, 0.03) |  |
| Range | 0.00 - 2.19 | 0.00 - 1.95 |  | 0.00 - 0.79 | 0.00 - 0.97 |  |
| **Eosinophils %** |  |  | 0.4902* |  |  | 0.9885* |
| N-Miss | 22 | 4 |  | 19 | 1 |  |
| Mean (SD) | 0.01 (0.02) | 0.01 (0.02) |  | 0.00 (0.01) | 0.01 (0.01) |  |
| Median     (Q1, Q3) | 0.00  (0.00, 0.01) | 0.00  (0.00, 0.01) |  | 0.00  (0.00, 0.00) | 0.00  (0.00, 0.00) |  |
| Range | 0.00 - 0.27 | 0.00 - 0.25 |  | 0.00 - 0.12 | 0.00 - 0.07 |  |
| **Monocytes** |  |  | 0.253* |  |  | 0.1643* |
| N-Miss | 23 | 4 |  | 19 | 1 |  |
| Mean (SD) | 0.56 (0.68) | 0.55 (0.32) |  | 0.69 (3.32) | 0.58 (0.41) |  |
| Median     (Q1, Q3) | 0.47  (0.32, 0.68) | 0.49  (0.33, 0.68) |  | 0.41  (0.25, 0.63) | 0.48  (0.27, 0.77) |  |
| Range | 0.01 - 23.31 | 0.02 - 2.45 |  | 0.02 - 66.34 | 0.07 - 2.01 |  |
| **Monocytes %** |  |  | 0.6814* |  |  | 0.2654* |
| N-Miss | 22 | 4 |  | 19 | 1 |  |
| Mean (SD) | 0.08 (0.04) | 0.08 (0.04) |  | 0.07 (0.05) | 0.07 (0.05) |  |
| Median     (Q1, Q3) | 0.07 (0.05, 0.10) | 0.07 (0.05, 0.10) |  | 0.06 (0.04, 0.08) | 0.06 (0.04, 0.09) |  |
| Range | 0.00 - 0.70 | 0.01 - 0.31 |  | 0.01 - 0.72 | 0.01 - 0.27 | p value |
| **Ferritin F** | **613 patients**  **(82.39%)** | **240 patients**  **(90.23%)** | 0.4706* | **131 patients**  **(17.61%)** | **26 patients**  **(9.77%)** | 0.3985* |
| N-Miss | 158 | 43 |  | 34 | 5 |  |
| Mean (SD) | 674.53 (817.61) | 564.63 (526.39) |  | 1237.07 (2308.64) | 2006.00 (4680.23) |  |
| Median     (Q1, Q3) | 459.00  (212.00, 820.50) | 433.00  (216.00, 750.00) |  | 700.00  (353.00, 1347.00) | 510.00  (269.00, 722.00) |  |
| Range | 4.00 - 7687.00 | 11.00 - 3397.00 |  | 19.00 - 20572.00 | 81.00 - 20941.00 |  |
| **Ferritin M** | **1070 patients**  **(78.56%)** | **354 patients**  **(90.23%)** | ***< 0.001**** | **292 patients**  **(21.44%)** | **56 patients**  **(9.77%)** | 0.1555* |
| N-Miss | 257 | 50 |  | 96 | 5 |  |
| Mean (SD) | 1353.00 (1359.86) | 1181.95 (3295.92) |  | 1825.25 (1945.47) | 1372.04 (1258.14) |  |
| Median     (Q1, Q3) | 939.00  (461.00, 1705.00) | 737.50  (405.25, 1283.00) |  | 1262.50  (572.25, 2323.25) | 1159.00  (598.00, 1500.00) |  |
| Range | 23.00 - 11513.00 | 25.00 - 56039.00 |  | 55.00 - 13289.00 | 112.00 - 7058.00 |  |

In bold and italics p-values<0.05

* Wilcoxon rank-sum test t

** Fisher’s exact test

**Supplementary File 1c**

| **Metrics** | **RF on a dataset rebalanced with the**  **SMOTE methodology** | | **RF on the original dataset** | |
| --- | --- | --- | --- | --- |
|  | **Training**  **March-April (MA)**  **(95% CI)** | **Validating**  **March-April (MA)**  **(95% CI)** | **Training**  **March-April (MA)**  **(95% CI)** | **Validating**  **March-April (MA)**  **(95% CI)** |
| **AUC** **(DeLong)** | 0.97  (0.97-0.98) | 0.83  (0.80-0.87) | 0.84  (0.82-0.86) | 0.82  (0.81-0.88) |
| **Sensitivity** | 0.93  (0.91-0.97) | 0.82  (0.72-0.92) | 0.81  (0.76-0.85) | 0.79  (0.66-0.95) |
| **Specificity** | 0.92  (0.88-0.94) | 0.75  (0.63-0.83) | 0.76  (0.73-0.79) | 0.76  (0.57-0.86) |

**Supplementary File 1d**

| **Metrics** | **LDH** | | | **D-dimer** | | |
| --- | --- | --- | --- | --- | --- | --- |
|  | **Training MA** | **Validating**  **MA** | **Testing**  **MD** | **Training MA** | **Validating**  **MA** | **Testing**  **MD** |
| **AUC (DeLong)**  **(95% CI)** | 0.95  (0.94-0.96) | 0.59  (0.54-0.65) | 0.55  (0.49-0.62) | 0.93  (0.92-0.94) | 0.56  (0.51-0.61) | 0.48  (0.40-0.56) |
| **Sensitivity**  **(95% CI)** | 0.89  (0.76-0.92) | 0.62  (0.53-0.71) | 0.67  (0.32-0.99) | 0.78  (0.75- 0.81) | 0.91  (0.50-0.96) | 0.48  (0.09-0.98) |
| **Specificity**  **(95% CI)** | 0.84  (0.80-0.96) | 0.61  (0.55-0.66) | 0.50  (0.11-0.80) | 0.94  (0.93- 0.96) | 0.26  (0.21-0.66) | 0.64  (0.04-0.95) |
| **Metrics** | **Neutr/Lymph** | | | **Neutrophils %** | | |
|  | **Training MA** | **Validating**  **MA** | **Testing**  **MD** | **Training MA** | **Validating**  **MA** | **Testing**  **MD** |
| **AUC (DeLong)**  **(95% CI)** | 0.73  (0.71-0.75) | 0.61  (0.56-0.66) | 0.63  (0.56-0.70) | 0.97  (0.97-0.98) | 0.52  (0.46-0.58) | 0.55  (0.49-0.62) |
| **Sensitivity**  **(95% CI)** | 0.63  (0.55-0.73) | 0.76  (0.50-0.93) | 0.52  (0.36-0.72) | 0.90  (0.86- 0.94) | 0.51  (0.09- 0.89) | 0.68  (0.36- 0.96) |
| **Specificity**  **(95% CI)** | 0.75  (0.65-0.82) | 0.48  (0.28-0.70) | 0.77  (0.55-0.84) | 0.94  (0.89- 0.96) | 0.59  (0.17- 0.96) | 0.50  (0.15- 0.77) |
| **Metrics** | **Fibrinogen** | | | **CRP** | | |
|  | **Training MA** | **Validating**  **MA** | **Testing**  **MD** | **Training MA** | **Validating**  **MA** | **Testing**  **MD** |
| **AUC (DeLong)**  **(95% CI)** | 0.89  (0.88-0.91) | 0.53  (0.48-0.59) | 0.49  (0.42-0.56) | 0.98  (0.98-0.99) | 0.57  (0.51-0.62) | 0.55  (0.48-0.62) |
| **Sensitivity**  **(95% CI)** | 0.64  (0.60- 0.84) | 0.54  (0.16- 0.93) | 0.5811  (0.05-1.00) | 0.91  (0.86- 0.96) | 0.43  (0.25- 0.69) | 0.56  (0.13- 0.88) |
| **Specificity**  **(95% CI)** | 0.97  (0.75- 0.98) | 0.59  (0.18- 0.91) | 0.50  (0.03- 0.99) | 0.94  (0.89- 0.98) | 0.74  (0.46- 0.88) | 0.62  (0.21- 0.95) |
| **Metrics** | **Brescia chest xray** | | | **Lymphocites %** | | |
|  | **Training MA** | **Validating**  **MA** | **Testing**  **MD** | **Training MA** | **Validating**  **MA** | **Testing**  **MD** |
| **AUC (DeLong)**  **(95% CI)** | 0.84  (0.82-0.86) | 0.63  (0.58-0.69) | 0.65  (0.59-0.72) | 0.95  (0.94-0.96) | 0.57  (0.51-0.62) | 0.61  (0.54-0.67) |
| **Sensitivity**  **(95% CI)** | 0.59  (0.53- 0.68) | 0.72  (0.50- 0.84) | 0.68  (0.56- 0.82) | 0.84  (0.80- 0.87) | 0.78  (0.22- 0.97) | 0.47  (0.22- 0.84) |
| **Specificity**  **(95% CI)** | 0.95  (0.88-1.00) | 0.52  (0.39- 0.72) | 0.63  (0.50- 0.70) | 0.93  (0.89- 0.96) | 0.37  (0.15- 0.91) | 0.75  (0.35- 0.94) |
| **Metrics** | **Ferritin std** | | | **Monocytes %** | | |
|  | **Training MA** | **Validating**  **MA** | **Testing**  **MD** | **Training MA** | **Validating**  **MA** | **Testing**  **MD** |
| **AUC (DeLong)**  **(95% CI)** | 0.94  (0.93-0.95) | 0.53  (0.48-0.58) | 0.55  (0.48-0.62) | 0.91  (0.90-0.92) | 0.51  (0.46-0.57) | 0.51  (0.44-0.57) |
| **Sensitivity**  **(95% CI)** | 0.76  (0.71- 0.82) | 0.92  (0.24-1.00) | 0.71  (0.54- 0.88) | 0.73  (0.69- 0.78) | 0.67  (0.09- 0.97) | 0.53  (0.05- 0.95) |
| **Specificity**  **(95% CI)** | 0.97  (0.91- 0.99) | 0.21  (0.1- 0.87) | 0.49  (0.31- 0.60) | 0.96  (0.91- 0.98) | 0.44  (0.11- 0.96) | 0.59  (0.11- 0.98) |

**Supplementary File 1e**

| **Biomarker** | **Threshold - Youden index**  **(95% CI)** |
| --- | --- |
| **LDH** | 268.20  (251.10-296.10) |
| **D-dimer** | 504.00  (504.00-524.60) |
| **Neutr/Lymph** | 4.56  (3.86-4.93) |
| **Neutrophils %** | 4.68  (3.68-6.16) |
| **Fibrinogen** | 417.50  (352.10-487.10) |
| **CRP** | 45.32  (37.42-124.40) |
| **Brescia chest xray** | 7.02  (6.06-8.01) |
| **Lymphocites %** | 1.01  (0.68-1.24) |
| **Ferritin std** | 14.24  (14.24- 14.27) |
| **Monocytes %** | 0.49  (0.34- 0.60) |
